# Experiences of family caregivers of persons living with dementia with and without a smart- clothes assisted home nursing program during the heightened COVID-19 alert

**DOI:** 10.1186/s12877-022-03379-8

**Published:** 2022-08-23

**Authors:** Ya-Li Sung, Huei-Ling Huang, Chung-Chih Lin, Teppo Kröger, Wen-Chuin Hsu, Jung-Lung Hsu, Yueh-E. Lin, Yea-Ing L. Shyu

**Affiliations:** 1 School of Nursing, Change Gung University, Guishan District, 259 Wenhua 1st Road, Taoyuan, 33302 Taiwan, ROC; 2 Department of Gerontology and Health Care Management, College of Nursing, Chang Gung University of Science and Technology, Taoyuan, Taiwan, ROC; 3 Geriatric and Long-Term Care Research Center, Chang Gung University of Science and Technology, Taoyuan, Taiwan, ROC; 4 Dementia Center, Department of Neurology, Taoyuan Chang Gung Memorial Hospital, Taoyuan, Taiwan, ROC; 5 Department of Computer Science and Information Engineering, Chang Gung University, Taoyuan, Taiwan, ROC; 6 Department of Social Sciences and Philosophy, University of Jyväskylä, Jyväskylä, Finland; 7 Centre for Care Research West, Western Norway University of Applied Sciences, Bergen, Norway; 8 Department of Neurology, New Taipei Municipal Tu Cheng Hospital, Chang Gung Memorial Hospital and Chang Gung University, New Taipei City, Taiwan, ROC; 9 Graduate Institute of Humanities in Medicine and Research Center for Brain and Consciousness, Taipei Medical University Shuang Ho Hospital, Taipei, Taiwan, ROC; 10 Department of Nursing, Linkou Chang Gung Memorial Hospital, Taoyuan, Taiwan, ROC; 11 Healthy Aging Research Center, Chang Gung University, Taoyuan, Taiwan, ROC; 12 Department of Nursing, Kaohsiung Chang Gung Memorial Hospital, Kaohsiung, Taiwan, ROC

**Keywords:** Smart-clothes, Homecare nursing, Family caregivers, Dementia, COVID-19 pandemic

## Abstract

**Background:**

The COVID-19 pandemic has required restrictions of daily activities, which has been found to impact the lives of persons living with dementia (PLWDs) and their family caregivers, who have multiple care demands. The lack of relevant studies in Taiwan emphasized the need to explore the experiences of family caregivers of older PLWDs faced with the intensified restrictions to control the spread of COVID-19, and the impact of the availability of a smart-clothes home nursing program.

**Methods:**

This qualitative study used semi-structured interviews with family caregivers of older PLWDs. Participants were recruited from dementia clinics of a medical center in northern Taiwan from a subset of a sample from a larger study on smart-clothes assisted home nursing care. A total of 12 family caregivers who participated in the original study were interviewed during the follow-up period; seven family caregivers of a PLWD wearing a smart-vest, which transmitted information to a home care nurse; five caregivers of a PLWD not wearing a smart-vest. Interviews were conducted by telephone because the conditions of the pandemic prevented face-to-face interviews. Recorded interviews were transcribed and analyzed using content analysis.

**Results:**

Interview data showed family caregivers’ felt the care recipient’s health was compromised and functional conditions intensified as Covid-19-related pandemic restrictions increased. Specific concerns included a lack social interactions, decreased daily activity levels, loss of interest and lack of motivation for activities, increased mood and behavioral problems, a decline in physical function and an increase in health problems. Family caregivers were also impacted by these restrictions, with significant increases in severity of caregiver role strain, including feeling trapped, a lack of in-home support, profound powerlessness, and worries about the PLWD contracting the coronavirus. The smart-clothes assisted home nursing care program offered supplementary support to family caregivers by providing on-time interactions, helping them manage health problems, enhancing predictability of the care recipient’s behaviors, and providing caregivers with emotional support.

**Conclusions:**

The findings of this study support alternative care such as implementation of technology-assisted home health services to meet caregiver needs to facilitate family caregiving of PLWDs during the necessary restrictions in activities implemented during the COVID-19 pandemic.

**Trial registration:**

ClinicalTrials.gov Protocol Record NCT05063045.

## Background

Dementia affects a large proportion of the global population. Over 50 million people worldwide are currently living with dementia, and the number is estimated to reach 82 million by 2030 and 152 million by 2050 [1]. In Taiwan, 7.98% of the population 65 years of age and older live with dementia [2], which will increase from 300,000 in 2020 to 460,000 in 2041, and 800,000 in 2061 [2]. Over 80% of persons living with dementia (PLWDs) in Taiwan are cared for by family members [3], which emphasizes the importance of family caregivers (FCGs) in this population. These caregivers experience high levels of caregiver burden, which has been associated with depression, lack of sleep and adverse outcomes to their health [4–9]. The current COVID-19 pandemic and the restrictions on daily activities required to control the spread of the virus is an additional impact on the lives of FCGs and PLWDs.

A review of 15 global quantitative demonstrated COVID-19 restrictions have had a detrimental effect on cognitive, behavioral, and neuropsychiatric symptoms along with declines in functional levels of activities of daily living (ADLs) for PLWDs [10]. A qualitative study by Sriram et al. (2021) found that COVID-19 restrictions also had a negative impact of caregivers of PLWDs in the UK, which included changes in their daily routines and reductions in wellbeing, social support, and respite from caregiving [11]. Another qualitative study in the UK called, “Promoting Activity, Independence and Stability in Early Dementia (PrAISED)” interviewed PLWDs, FCGs, and therapists in May of 2020 and again in July of 2020 [12]. Participants reported the lock-down restrictions from COVID-19 caused PLWDs to feel a lack of motivation and a reduction in cognitive abilities to maintain daily activities. The added requirements of maintaining social distancing and cessation of daily outdoor activities have caused a reduction in physical activity, social participation, and a lack of cognitive stimulation[13–15]. The COVID-19 pandemic has widened the differences between existing care methods and typical challenges and difficulties experienced by FCGs, which has led to increased caregiver burden related to practical difficulties, emotional stress, and difficulty asking for help [11–13, 16, 17].

Prior to May 2021, few cases of COVID-19 had been reported in Taiwan due to high border control restrictions. However, following a domestic outbreak of cases in May 2021, the number of daily confirmed COVID-19 cases increased rapidly from single digits to hundreds and by May 18, 2021, the Taiwan Central Epidemic Command Center (CECC) raised the COVID-19 alert to Level 3 [18], which continued until July 26, 2021, when the outbreak had subsided. The period of intensified restrictions required everyone to wear a mask “at all times” when outside the home, avoid unnecessary activities and gathering, and importantly, community and in-home long-term care services were unavailable and day care centers and local dementia centers were closed [19]. However, there is a lack of studies related to the impact of these COVID-19 restrictions on the caregiving experiences for FCGs of PLWDs in Taiwan.

COVID-19 restrictions have generated unprecedented challenges for FCGs and PLWDs, especially in the reorganization of their daily routine and care relationships [13]. These caregivers have multiple needs which are more difficult to address during restrictions of the COVID-19 pandemic, which may need what Rokstadt et al. (2021) refer to as “radical changes” in services, such as a greater availability of telemedicine for consultations with dementia specialists and social support services either via video- or telephone-based services [17]. An interventional study by Lai et al. (2020) also found that telemedicine by video conference during the COVID-19 pandemic was associated with improved resilience and wellbeing for both persons living with dementia and their home caregivers [20]. 

One source of meeting the needs of caregivers of PLWDs during pandemic restrictions could be the availability of a smart-clothes home nursing care model, which merges smart technology and wearable sensors to provide remote health care and assist family caregivers in providing care to older persons with physical or cognitive impairment in a home setting [21, 22]. This technology could help FCGs of PLWDs facilitate balancing care responsibilities and other demands such as employment and household responsibilities. This smart-clothes home nursing care model monitors the number of steps taken as a measure of daily activity, location of the person in the home, number of times getting up at night, posture, and leaving the home. Caregivers are provided with information about the care recipient’s activities through reports from a nurse supervisor, who also acts as a reference for enhancing caregiving and support. This care model allows family caregivers to have a better understanding of the recipient’s daily activities, including attempts to leave the home unsupervised, wandering, and getting up during the night, and feedback from a nurse researcher helped them design their caregiving tasks to target individual needs, enhance balance between competing needs, and decrease anxiety and depressive symptoms [22, 23].

The purpose of this study was to: 1) develop a general understanding of FCG experiences during the initial phase of the intensified Level 3 COVID-19 restrictions; and 2) examine differences and similarities between those who participated in a smart-clothes assisted home nursing care program and those who did not, and whether the nursing care program benefited those who participated.

## Methods

### Study design

This study used a qualitative descriptive design with individual semi-structured interviews with FCGs of older PLWDs. Data were collected from 19 July 2021 to 23 July 2021.

### Setting and participants

Data for this study were collected using follow-up interviews with a subset of caregivers participating in an on-going, long-term interventional study on the effects of a smart-clothes assisted home nursing care program. Participants were FCGs of older PLWDs recruited from dementia clinics of a medical center in northern Taiwan. The criteria for PLWDs were: (1) 60 years of age or older; (2) diagnosed with dementia by a neurologist and (3) living with at least one family member in northern Taiwan. Exclusion criteria were (1) presence of a psychiatric disorder; (2) terminally ill; (3) without a primary family caregiver, and (4) living in a long-term care facility. Criteria for FCGs included being at least 20 years of age and the primary person responsible for providing direct care or supervising the care received by the PLWD. Caregivers who agreed to participate in the study provided signed consent. Of 367 PLWDs screened in the dementia clinics, among the 109 who met the inclusion criteria, 26 dyads of FCGs and PLWDs agreed to participate. Twelve caregivers who had been participating remained in the study during the 2-month period of the heightened COVID-19 alert (May 18, 2021 to July 18, 2021) and were invited for follow-up interviews; seven were caregivers of PLWDs who wore the smart-vest (FCG + SV); five were caregivers of PLWDs who did not wear the smart-vest (FCG–SV); none of the invited participants had dropped out during the 2-month period.

### Smart clothes-assisted home-nursing care program

The smart-clothes assisted home nursing care program utilized a smart-vest, which was worn 24 h per day by PLWDs and contained sensors capable of detecting movement and activity of within the home environment in real time. The sensors were linked to movement detectors in bedrooms and living areas, and a special front-door-detector was used to alert any unintended departure from the home. Information from the sensors was based on smart technology and was immediately transmitted to an Application (App) on the home care nurse’s smart phone, who then provided feedback to FCGs to facilitate caregiving. An initiation period of accumulated data was used to establish threshold levels for typical activity levels, which allowed for subsequent detection and alerts of low or high activity that could be interpreted as atypical, such as getting up at night, staying in the bathroom for more than 30 min, not moving during the day for more than 2 h, and leaving the house unaccompanied. The home care nurse assigned to support the FCG and PLWD provided instant feedback in an emergency and weekly regular feedback to caregivers about managing care for the PLWD, which included encouraging daily activities, providing safety measures for getting up at night or going out, and managing the agitation of PLWDs based on the information and signals collected through the smart-vest.

### Data collection

These data were obtained from a small subset of participants enrolled part in our three-year clinical program. Qualitative data were collected with individual semi-structured interviews. Interviews followed an interview guide and were conducted by telephone because the conditions of the pandemic prevented face-to-face interviews.

One-on-one telephone interviews were conducted by three trained and experienced gerontological nurses: a doctoral candidate (YLS), and two research assistants with more than 10 years’ experience with dementia nursing care (PLS and YTW). Interviews were conducted at a time chosen by the caregivers. Prolonged engagement with the participants over the previous month prior to the COVID-19 restrictions allowed the researchers to establish close relationships, thus participants were more willing to express their feelings and experiences. Interviews were tape recorded with the participant’s permission and each lasted around 20–30 min. Sample questions for FCGs included: “Can you describe what you must do to care for your husband/mother during a typical day?”; “How did these experiences differ following the announcement of the Level 3 alert by the CECC?” For those FCGs who participated in the smart-clothes assisted home nursing care program, we additionally asked “What was your experience with the smart-clothes program?”; “In what ways has the smart-clothes nursing care program been helpful or not in your caregiving?” During the interviews field notes were maintained by the researchers.

Quantitative data for PLWDs were also collected regarding ADLs and scores on the Mini-Mental State Examination (MMSE). These data were collected when the Level 3 COVID-19 alert was implemented and just prior to the interviews (approximately 2 months later), to evaluate any changes in cognitive function and ADLs in PLWDs during this period that might support the subjective experiences reported by participants.

### Ethical considerations

The original study was conducted after approval of the human subject research from the study hospital ethics committee (Chang Gung Medical Foundation, Institutional Review Board; approval number: No. 20170206B0). Data were collected after the participants provided signed informed consent.

### Data analysis

Tape recorded interviews were transcribed and interview transcripts and field notes were analyzed using content analysis [24] for the qualitative portion of the study. The two researchers listened to the taped interviews, read the transcripts, and performed open coding and content analysis, in line with standard research procedures of Miles and Huberman (1994), using three types of codes: descriptive, interpretative, and pattern [25]. Descriptive codes require little interpretation. For example, when a family caregiver said, *‘*“*If she goes to the center to exercise with other people, she is more motivated, but when she stays home, she loses her motivation*”, *w*e coded the statement as ‘loss of interest’ based on the family caregiver’s words. Interpretive codes involve the researcher’s interpretation. For example, one family caregiver said,

*Before the Level 3 COVID-19 alert, I only needed to be with my mom during weekdays, but now, I must stay with her every day! I cannot do anything I need to do or have my own space during the daytime, and I must watch out for her all the time.* This statement was coded as ‘being trapped’.

After the initial summarizing of segments of data using interpretive descriptions, data were transformed into pattern codes. Pattern codes that represent emergent themes and categories are more inferential and explanatory and can capture patterns appearing in the data. Thus, pattern codes were grouped and condensed into categories or themes. For example, *‘*aggravated family caregiver role strain*’* was coded as a pattern describing the caregiver’s experiences and feelings, which included the feelings of being trapped, profound powerlessness, lack of support, and worries caused by measures of the raised alert for Covid-19 prevention. The coding process was completed independently by the first author and the corresponding author, who discussed, verified, and clarified the interview data to establish a mutually agreed upon code. During the analysis, we used both within- and across-case analyses. Quantitative data including ADLs and MMSEs were presented as means and standard deviations.

### Rigor

Trustworthiness of this qualitative study was ensured by four criteria: credibility, dependability, transferability, and confirmability [26]. Credibility of the data was enhanced by the researchers’ prior interactions with PLWDs and their FCGs by providing home nursing care to the families in the community before the interviews, which promoted prolonged engagement. Dependability was ensured by maintaining an audit trail of the data, including reflexive journals of ideas and thoughts, which was examined by a panel of experts in dementia care, qualitative research, gerontological nursing, and information technology. Confirmability was enhanced through peer debriefing with two gerontological nurse experts and two qualitative method experts, who reviewed the fit of the raw data and themes, triangulation and member checks with family caregivers to ensure interpretations of the interview data reflected the participants’ experiences. Finally, transferability of the data was established by rich descriptions of contextual conditions of the phenomena [26].

## Results

### Demographic backgrounds

Characteristics of the 12 FCGs who participated in the follow-up interviews and the PLWDs are shown in Table 1. The mean age of the FCGs was 62 years (SD = 9.83); eight were college educated. The relationships between the caregivers and PLWDs were comprised of spouses (wives, n = 4), and adult children [sons (*n* = 2) and daughters (*n = *6)]. Eight FCGS received outside caregiving support, which included support from day care centers (*n *= 2), homecare services (*n = *4), caregiver support groups (*n* = 3) and community care centers (*n = *5). The mean age of PLWDs was 82.83 years (SD = 8.07); seven were female; and eight had an educational level of high school or above. Three PLWDs had mild cognitive impairment (CDR = 0.5) and nine were diagnosed with moderate dementia (CDR = 1). Four FCGs who continued with the smart-clothes program after the Level 3 alert were wives; one son and two daughters were caregivers for their father. All caregivers of PLWDs who did not wear the smart-vest (PLWDs-SV) were adult children of their mother (one son and four daughters).

**Table 1:** Characteristics of family caregivers and persons with dementia (*N* = 12 dyads)

	Overall		With Smart vest		Without Smart vest	
	(*N* = 12)		(*n* = 7)		(*n* = 5)	
**Characteristic**	***n* (%)**	Mean ± SD	***n* (%)**	Mean± SD	***n* (%)**	**Mean± SD**
**Family caregivers**						
Age (years)		62.00 ± 9.83		66.43 ± 10.03		55.80 ± 57.1
Gender						
Female	10 (83.3)		6 (85.7)		4 (80.0)	
Male	2 (16.7)		1 (14.3)		1 (20.0)	
Education						
Elementary	1 (8.3)		1 (14.3)		0 (0)	
Junior high	0 (0)		0 (0)		0 (0)	
High school	3 (25.0)		3 (42.9)		0 (0)	
University	8 (66.7)		3 (42.9)		5 (100)	
Relationship						
Spouse (wife)	4 (33.3)		4 (57.1)		0 (0)	
Adult child^a^	8 (66.7)		3 (42.9)		5 (100)	
Living with care recipient						
No	2 (16.7)		0 (0)		2 (40.0)	
Yes	10 (83.3)		7 (100)		3 (60.0)	
Outside care support						
No	4 (33.3)		4 (57.1)		0 (0)	
Yes	8 (66.7)		3 (42.9)		5 (100)	
Type of outside support						
Day care center	2 (16.7)		1 (14.3)		1 (20.0)	
Home care	4 (33.3)		3 (42.9)		1 (33.3)	
Caregiver support group	3 (25.0)		2 (28.6)		1 (20.0)	
Community care center	5 (41.7)		1 (14.3)		4 (80.3)	
**Persons with dementia**						
Age (years)		82.83 ± 8.07		82.57 ± 8.99		83.20 ± 7.59
Gender						
Female	7 (58.3)		2 (28.6)		5 (100)	
Male	5 (41.7)		5 (71.4)		0 (0)	
Education						
Elementary	3 (25.0)		2 (28.6)		1 (20.0)	
Junior high	1 (8.3)		1 (14.3)		0 (0)	
High school	4 (33.3)		2 (28.6)		2 (40.0)	
University	4 (33.3)		2 (28.6)		2 (40.0)	
CDR score						
0.5	3 (25.0)		2 (28.6)		1 (20.0)	
1	9 (75.0)		5 (71.4)		4 (80.0)	

*Note:* Mean mean, *SD* Standard deviation, *CDR* Clinical dementia rating

^a^Two sons, six daughters

### Overall experiences of family caregivers

Similar experiences were found for family caregivers who participated in the smart-clothes assisted home nursing care program and those who did not for the two main themes of “Alterations in social and behavioral functions” and “Aggravated family caregiver role strain”. Each theme’s corresponding categories are described below.

### Alterations in social and behavioral functions

Analysis of interview data obtained 2-months after the Level-3 alert indicated most FCGs believed PLWDs experienced a decline in health and physical function. They reported PLWDs exhibited reduced social interactions, a decrease in daily activities, loss of interest and a lack of motivation for activities, increased mood and behavioral problems, increased health problems and a decline in physical function. These categories were identified by all participants regardless of whether they did or did not receive the smart-clothes assisted home nursing care program. All participants reported alterations in social and behavioral functions of PLWDs.

#### Reduced social interactions

Many caregivers reported the care recipient ceased participating in their usual extracurricular activities. Six FCGs mentioned PLWDs were unable to attend day care facilities or activity centers. One wife caregiver commented, *“He used to have some good friends; he would go out and socialize with them. It was good for him, but now he is prevented from doing that.”* (FCG28 + SV). A daughter caregiver whose mother did not wear the smart-vest shared a similar experience saying, *“She stayed home without outside stimulus, without contacting other people and no one comes to visit.”* (FCG42–SV).

#### Decreased activity level

Nearly all caregivers mentioned a decrease in the daily activity of PLWDs following the Level-3 alert. This subjective perception of inactivity was confirmed with quantitative data from the smart-vests worn by PLWD who continued to participate in the program. For instance, the average daily steps for PLWD29 + SV decreased from 2538 steps/day to 1968 steps/day following the alert, a decrease of 570 steps/day. The daily steps for PLWD31 + SV decreased from an average of 4000–5000 steps a day to 2000 steps/day, a decrease of between 2000 to 3000 daily steps; PLWD32 + SV went from 2096 steps/day to 1647 steps/day during the first two months, a decrease of 837 daily steps. One daughter caregiver reported a significant change in her mother’s daily schedule:

*Before the Level 3 COVID-19 alert, my mother used to get up at 8 am, but since the Level 3 COVID-19 alert, she now sleeps until noon; she used to eat three meals a day, now she eats only two meals a day.* (FCG29 + SV).

#### Loss of interest and lack of motivation

All family caregivers reported PLWDs exhibited a loss of interest in their usual daily activities and a lack of motivation for exercising, regardless of whether the PLWD wore a smart-vest. One son caregiver said, “*If she goes to the center to exercise with other people, she is more motivated, but now she must stay home and has lost her motivation*.” (FCG32 + SV). A daughter caregiver whose mother did not wear a smart-vest said, “*She used to like to go out with me for a walk. Now, she does not want to go and says, ‘You go by yourself’”* (FCG33 + SV). One caregiver in the smart-vest program said *“After the Level 3 COVID-19 alert, she does not even want to walk, sometimes I ask her to get up and do the exercise film you* [the home care nurse] *sent, but she will not do it. She said, ‘you can watch the film yourself’”* (FCG28 + SV)*.*

#### Increased mood and behavioral problems

Mood and behavioral changes among the PLWDs varied over the 2-month period. Caregivers of PLWDs who did not wear the smart-vest reported more behavioral problems than caregivers in the smart-clothes support program. One of these FCGS, a daughter caregiver (FCG33–SV) said, *“She used to smile when she interacted with you or wanted to talk, but now she looks apathetic and expressionless, and she is very quiet.”* Another daughter caregiver, whose mother did not wear the smart-clothes said,

*Before [the alert], she did not want to take a shower. But after the Level 3 COVID-19 alert, she did not even want to change her clothes.*.. *now she gets up in the middle of the night and asks our maid to cook or says someone is calling from outside the door*. *(FCG42*–SV).

In the group who cared for a PLWD who wore the smart-vest, only one caregiver (FCG37 + SV) reported the PLWD (her husband) had increased behavioral problems. This was because during the first month of the Level 3 COVID-19 alert he became irritable because he did not understand why he and his wife could no longer go out. The wife caregiver was able to receive guidance through the smart-clothes assisted nursing care, and the husband’s behavioral problems improved.

#### Functional decline and health problems

Most of the PLWD were found to have memory and physical function declined after the Level 3 COVID-19 alert. Assessments on the MMSE before and 2-months after the alert showed a decline in scores for eight PLWDs. Family caregivers attributed the decline to lack of participation in social and community services, lack of cognitive stimulus and activities. A daughter caregiver described the decline of her mother saying, “her reaction has become slower and her memory is worse. She used to be able to recall her children’s names, now she is unable to remember them, even if she sees them.” (FCG29 + SV). A son caregiver said, *“During this period of time, I found as her activity decreased, she regressed and her physical function became poorer.”* (FCG32 + SV*).*

Similar problems were reported by caregivers not receiving smart-clothes support. A daughter caregiver (FCC33–SV) mentioned her mother’s reactions were slower and short-term memory became poorer. Five family caregivers expressed worries and concerns regarding further physical and cognitive function decline they believed were due to a reduction in social interactions after the Level 3 COVID-19 alert (FCG28 + SV, FCG30 + SV, FCG35 + SV, FCG38–SV, FCG39–SV). During the Level 3 COVID-19 alert, other health management problems also occurred. One daughter caregiver not receiving smart-clothes support described her mother’s inability to continue to manage her diabetes:

*She has been giving herself insulin injections for over 10 years and it has been a very easy and routine task for her. Suddenly, she has forgotten how to do it. She forgets to take her medication too, which might cause her blood sugar to be too low or too high. It is very dangerous.* (FCG33–SV*).*

FCGs’ perceptions of functional decline of the PLWDs were also supported by routine clinical measures of the MMSE and ADLs (Table 2).

**Table 2:** Cognitive function and activities of daily living scores for persons with dementia (*N *= 12) pre-and post-Level 3 COVID-19 alert

	**Pre-alert**	**Post-alert**
**Measure**	Mean ± SD	Mean ± SD
MMSE, overall score		
All care recipients (*N* = 12)	22.25 ± 3.54	19.10 ± 6.21
With smart vest (*n* = 7)	22.04 ± 4.32	19.58 ± 7.27
Without smart vest (*n* = 5)	22.60 ± 2.50	18.42 ± 6.76
ADLs		
All care recipients (*N* = 12)	92.53 ± 8.03	90.12 ± 8.66
With smart vest (*n* = 7)	93.00 ± 9.74	92.00 ± 9.08
Without smart vest (*n* = 5)	92.72 ± 8.35	90.90 ± 8.47

*Note: SD* Standard deviation, *MMSE* Mini-mental state examination, *ADLs* Barthel’s index for activities of daily living

### Aggravated family caregiver role strain

The experience of aggravated family caregiver role strain after the Level 3 alert included four categories: being trapped, lack of in-home support, profound powerlessness, and increased worry about the PLWD contracting the virus. These categories were described by all participants with and without the home nursing care program.

#### Being trapped

Several caregivers with or without smart-clothes support expressed their feeling of being trapped. One daughter caregiver, whose mother wore the smart-vest said,

*Before the Level 3 COVID-19 alert, I only needed to be with my mom during weekdays; now, I must stay with her every day. I cannot do anything I need to do or have my own space during the daytime and I must watch out for her all the time. (*FCG29 + SV*).*

#### Lack of in-home support

In-home services were a significant complaint of all caregivers, with or without smart-clothes support. One wife caregiver whose husband wore the smart-vest said, *“The home aide has not been able to come for two months, he has no one to take him out.*” (FCG31 + SV). A son caregiver complained about the loss of in-home rehabilitation support for his mother saying, *“We used to have someone come in to help her do some cognitive exercise or rehabilitation, but now, these activities were stopped.”* (FCG32 + SV).

#### Profound powerlessness

Family caregivers expressed feeling powerlessness, which was aggravated by the additional time spent with the PLWD during the Level 3 COVID-19 alert compared with before the alert. One daughter caregiver with no smart-clothes support said:

*Before, I always felt that as long as I put effort into caregiving, my mom would have some improvement. Now, after spending much more time with her, whatever you ask her, she says she does not know. It is so difficult to communicate with her. I really want to know whether she can still think or not. I am very frustrated. I need to get out of this negative feeling and give myself more time to interact more positively with her.* (FCG33–SV*).*

A wife caregiver with smart-clothes support said that: *“He is too lazy, if he keeps doing this, I am going to give up on him. We want to help him, but he is so passive and not wanting to do anything. I am so mad. I told my daughter and grandson, ‘let him do it by himself, don’t help him’.” (*FCG37 + SV*).*

#### Worry about COVID-19 infection

Most caregivers worried about the PLWD contracting COVID-19. One daughter caregiver with no smart-clothes support and who did not reside with her mother described trying to reduce the risk to her mother:

*I go home every day to check if she and the other members of the family are okay. When I go home, I always wore a mask and did not dare to eat with them. I only brought fresh food for them and would not say long.* (FCG42–SV).

A son caregiver with no smart-clothes support said*, “If anyone wants to come visit her, we take their temperature, and use alcohol to sanitize before coming into our house.”(*FCG38–SV*)* One daughter caregiver said, “*My mom does not remember anything, I told her not to go out, there are COVID virus out there. She asked me, ‘what is that?’ I have to keep explaining (what COVID is) to her.” (*FCG40–SV*).*

### Smart-clothes experiences: Supplementary support from Smart-clothes assisted home nursing

During the two months of the Level 3 alert, seven of the caregivers continued to receive smart-clothes assisted home nursing. These seven FCGs reported several benefits provided by this care model, which were due to the supplemental support from this program. The following categories were identified only by those FCGs who received the smart-clothes assisted home nursing care: on-time social interactions, management of health problems, enhanced caregiving predictability, and emotional support.

#### On-time social interactions

One family caregiver appreciated support via exchanges over video calls from the home care nurse, which provided social interactions and emotional support for the PLWD. This daughter caregiver said,

*I think it is very good that the home care nurse uses the video calls to teach her (my mother) how to exercise and to ask her how she is doing. You can see that her mood improves after she has talked to her (the home nurse) and she is more willing to exercise”* (FCG29 + SV).

#### Management of health problems

Smart-clothes assisted home nursing was able to help when accidents occurred during the Level 3 alert. One home care nurse noticed a significant drop in the level of activity transmitted from the smart-vest of one of the care recipients (PLWD29 + SV). The nurse immediately contacted the daughter caregiver (FCG29 + SV) and asked her to check on her mother, who had apparently fallen. This allowed the nurse to provide a timely intervention, which included assessments for post-fall conditions, consultations in the hospital, health education regarding fall prevention, and video conferencing for suggestions of modified activities and exercise coaching.

A son caregiver’s mother’s activity had declined, which was transmitted to the home care nurse. The nurse immediately consulted with the son and determined his mother’s inactivity was due to constipation. The nurse provided health education on constipation and encouraged more exercise, which resolved the problem. The son caregiver said, “*It was very helpful that through the smart-vest, the nurse could be notified of my mom’s situation, remind my mom to exercise and provide health education to help my mom improve her constipation.*” (FCG32 + SV).

#### Enhanced caregiving predictability

The home care nurse manager involved in the smart-clothes assisted home nursing program provided caregivers with information about the PLWD, which included changes in activity level, frequency of getting out of bed at night, and the time these events were most likely to occur. The family caregivers reported this information was beneficial for facilitating their caregiving of the PLWD. One wife caregiver said,

*During the Level 3 COVID-19 alert, although the home care nurse was not able to come visit, she could give us information from the smart-vest, so we would know when he was getting out of bed at night. Now I can be more alert and attentive to his situation and prevent him from falling. I feel lucky to participate in this study.*” (FCG31 + SV).

A son caregiver described help he received for his mother: *“The home care nurse called and let us now that her activity level was really low, and she reminded and encouraged us to increase her exercise. That was very helpful.*” (FCG32 + SV).

#### Emotional support

Most caregivers in the smart-clothes program mentioned the home care nurses responded to their personal concerns and worries during the COVID-19 alert, which made FCGs feel supported. A son caregiver said, *“Although the home nurse cannot come* [to our house]*, she calls me or talks to me on-line. I am able to talk to her about my concerns, it really lessens my worries.”* (FCG32 + SV). A daughter caregiver said,

*When my mom fell that day, no one was home with us. I am so glad that the home nurse could tell me what I needed to do. Although these few days have been really stressful, the home nurse called and supported me, which made me feel more at ease and cared for.* (FCG29 + SV).

## Discussion

This study is the first to explore the impact of the COVID-19 Level 3 alert in Taiwan for caregivers and their family member with dementia. In addition, our study explored whether a smart-clothes home nursing care program had a positive effect on FCGs and PLWDs. This study provided empirical data from caregivers of PLWDs on decreased levels of activity and identified possible reasons for the decline, which included lack of social interactions, loss of interest and lack of motivation. Our findings of increased behavioral problems and functional decline reported by FCGs support prior studies on the effects on cognitive and physical health of PLWDs during COVID-19 restrictions in the UK [10, 12] as well as for persons with other dementias, such as Alzheimer’s disease [15]. Caregivers in our study reported the increased restrictions put a strain on the unmet needs of the existing care model in Taiwan and created challenges to the reorganization of daily routines and care relationships for family caregivers. These findings have also been reported for FCGs of PLWDs in Italy [16] and Norway [17], who reported COVID-19 restrictions increased caregiver burden due to difficulties in maintaining daily activities, increased emotional stress, and difficulty in reaching for help [16, 17].

Eight of the caregivers in our study received additional caregiving support services prior to the restrictions, such as home care, the use of day care centers, and support groups, which were not available when COVIC-19 restrictions took effect. This increased the amount of time FCGs and PLWDs interacted, and caregivers reported feeling trapped, having no emotional support, experiencing profound powerless, and worries about PLWDs contracting the virus. On the other hand, although most of the FCGs in our study were concerned about the PLWD being infected and attempted to protect the PLWD from exposure, none of the FCGs expressed worries regarding who would provide care for the PLWD if they themselves needed to be isolated or required hospitalization due to COVID-19. This lack of concern was also report by Vaitheswaran et al. (2020) for FCGs of PLWDs in India [27]. Family caregivers worry about the PLWD becoming ill might be due to the lower level of infection and severity of symptoms experienced by the general population in Taiwan compared with other countries.

Prior studies documented the effects of smart-home technologies on monitoring older care receivers’ behaviors and activities to guide caregiving safety actions [21, 22]. One study found that telehealth care combined with discharge planning decreased caregiver burden, improved mastery of stress and overall family function [4]. Our previous study found that smart-clothes-assisted home-nursing care program increased family caregivers’ knowledge of older care recipients’ condition, informed health care providers about the care recipient’s condition, helped the home care nurses provide timely interventions, balanced care recipients’ exercise and safety, helped family caregivers balance work outside the home and caregiving [28]_._The experiences reported by the FCGs in our study with a PLWD using the smart-vest showed loss of community and home services that occurred for the other FCGs due to the Level 3 alert was somewhat mitigated by the smart-clothes assisted home nursing program. The program provided PLWDs with on-time interactions, consultations for FCGs regarding management of health-related problems, enhanced predictability of caregiving needs, and emotional support for FCGs. Data transmitted from the smart-vest also provided quantitative confirmation of lower levels of activity for PLWDs 2 months after the COVID-19 restrictions were initiated. Family caregivers draw attention to the need for professional consultation through technology [22, 27, 29]. Although the smart-clothes assisted home nursing was not a substitute for services such as in-home nursing care or community activities, it was a supplement for health care needs of the PLWDs by facilitating interactions with FCGs and the home care nurses.

### Limitations

Our study had some limitations. Taiwan’s experience with COVID-19 is unique among most countries in the world because the general requirements for control of the virus since 2020 has kept case reported counts much lower per 100 000, and the Level 3 alert was not as stringent as the lockdowns in other countries. Second, in Taiwan, over 80% of PLWDs live with one or more family members, thus, transferability of the study results regarding the impact of COVID-19 restrictions should be interpreted with caution. Third, interviews were conducted only two months after the Level 3 alert was initiated; therefore, these findings do not reflect long-term effects of the restrictions. We suggest additional interviews be conducted in the future with this population of caregivers to better understand the long-term impact of the COVID-19 restrictions on FCGs and PLWDs in Taiwan. Finally, the small sample size and without random assignment makes the detection of the true effects of the smart-clothes assisted home nursing program difficult.

## Conclusions

Our findings provide empirical evidence on the influences of the COVID-19 Level 3 alert and the resulting restrictions on FCGs and PLWDs in Taiwan. The restrictions that were initiated compromised health and functional conditions for PLWDs, which included lack social interactions, decreased levels of daily activity level, loss of interest and lack of motivation for activities, increased mood and behavioral problems, declines in physical function and increased health problems. The restrictions also had an impact on the caregivers, which included increases in caregiver role strain due to feelings of being trapped, lack of in-home support, profound powerlessness, and worries about the PLWD contracting the virus.

Caregivers who were also participants in the smart-clothes assisted home nursing care program provided additional information on the benefits that this innovative program can provide during pandemic restrictions that limit access to supplementary support for FCGs and PLWDs. The smart-vest provided quantitative feedback on activity levels of PLWDs and the experiences of the FCGs support this care model as an intervention that can provide PLWDs on-time interactions to improve management of health problems, enhance FCGs caregiving abilities, and provide emotional support. FCGs faced some challenges, such as the reluctance of PLWDs to wear the smart-vest, the lack of a home network, and device installation issues, but these were solved one by one. An earlier study demonstrated the utility and acceptability or the smart-clothes system [28]. The findings of this study provide a basis for implementing technology assisted home health services during intensified control resulting from pandemic restrictions.

## Data Availability

The datasets generated and/or analyzed during the current study are not publicly available due to the reason that the formal study is still ongoing but are available from the corresponding author upon reasonable request.

## Abbreviations

ADLs: Activities of daily living
App: Application
CDR: Clinical dementia rating
CECC: Central epidemic command center
FCGs: Family caregivers
PLWDs: Persons living with dementia
MMSE: Mini-mental state examination
